# Replacing manual planning with automatic iterative planning for locally advanced rectal cancer VMAT treatment

**DOI:** 10.1002/acm2.14552

**Published:** 2024-10-15

**Authors:** Jiacheng Liu, Ruoxi Wang, Qingying Wang, Kaining Yao, Meijiao Wang, Yi Du, Haizhen Yue, Hao Wu

**Affiliations:** ^1^ Key laboratory of Carcinogenesis and Translational Research (Ministry of Education/Beijing), Department of Radiation Oncology Peking University Cancer Hospital & Institute Beijing China; ^2^ Institute of Medical Technology Peking University Health Science Center Beijing China

**Keywords:** automatic iterative planning, locally advanced rectal cancer, volumetric‐modulated arc therapy

## Abstract

**Purpose:**

To develop and implement a fully automatic iterative planning (AIP) system in the clinical practice, generating volumetric‐modulated arc therapy plans combined with simultaneous integrated boost technique VMAT (SIB‐VMAT) for locally advanced rectal cancer (LARC) patients.

**Method:**

The designed AIP system aimed to automate the entire planning process through a web‐based service, including auxiliary structure generation, plan creation, field configuration, plan optimization, dose calculation, and plan assessment. The system was implemented based on the Eclipse scripting application programming interface and an efficient iterative optimization algorithm was proposed to reduce the required iterations in the optimization process. To verify the performance of the implemented AIP system, we retrospectively selected a total of 106 patients and performed dosimetric comparisons between the automatic plans (APs) and the manual plans (MPs), in terms of dose‐volume histogram (DVH) metrics, homogeneity index (HI), and conformity index (CI) for different volumes of interest.

**Result:**

The AIP system has successfully created 106 APs within clinically acceptable timeframes. The average planning time per case was 36.8 ± 6.5 min, with an average iteration number of 6.8 (±1.1) in plan optimization. Compared to MPs, APs exhibited better performance in the planning target volume conformity and hotspot control (p<0.001). The organs at risk (OARs) sparing was significantly improved in APs, with mean dose reductions in the femoral heads, the bone marrow, and the SmallBowel‐Avoid of 0.53 Gy, 1.18 Gy, and 1.00 Gy, respectively (p<0.001). Slight improvement was also observed in the urinary bladder $V_{40\;\mathrm{Gy}}$ and the small bowel $D_{2\;\mathrm{cc}}\;\left(p<0.001\right)$. Additionally, quality variation between plans from different planners was observed in DVH metrics while the APs represented better plan quality consistency.

**Conclusion:**

An AIP system has been implemented and integrated into the clinical treatment planning workflow. The AIP‐generated SIB‐VMAT plans for LARC have demonstrated superior plan quality and consistency compared with the manual counterparts. In the meantime, the planning time has been reduced by the AIP approach. Based on the reported results, the implemented AIP framework has been proven to improve plan quality and planning efficiency, liberating planners from the laborious parameter‐tuning in the optimization phase.

## 1 INTRODUCTION

Rectal cancer is a common malignant cancer of the digestive system, with approximately 732,210 new cases and 339,022 deaths in 2020.^1^ Preoperative chemoradiotherapy is commonly employed as a neoadjuvant treatment for locally advanced rectal cancer (LARC), owing to its effectiveness in tumor invasion control and median survival improvement.^2^, ^3^, ^4^ During chemoradiotherapy, it is crucial to manage the hematologic and gastrointestinal toxicity, minimizing negative impacts on patient health and treatment effectiveness.^5^, ^6^ The development of new delivery techniques such as intensity‐modulated radiotherapy (IMRT) and volumetric‐modulated arc therapy (VMAT) has enabled the simultaneous integrated boost (SIB) technique to deliver varying doses to different target volumes in a single fraction.^7^ The SIB‐VMAT technique has been proven to be advantageous in rectal carcinoma treatment due to its superior organs‐at‐risk (OARs) sparing and tumor control.^8^, ^9^

Although modern computing hardware has significantly accelerated various processes in treatment planning, the overall time to generate a deliverable plan is still significant. The bottleneck of this slow process was the existence of multiple rounds of human‐machine interaction. In addition, the treatment plan quality varies due to the planner's experience or time pressure. Automating the treatment planning process is an ideal option to mitigate the aforementioned challenges in manual treatment planning, as several studies have shown that planning efficiency, quality, and consistency can be improved with automatic treatment planning.^10^, ^11^, ^12^, ^13^ There are two major types of automatic treatment planning approaches: knowledge‐based planning (KBP) and automatic iterative planning (AIP). The KBP leverages historical plans to build dose prediction tools and guides treatment planning on new patients with predictive dose‐volume histogram (DVH) metrics.^14^, ^15^ However, the performance of the KBP methods heavily depends on the quality and consistency of data used for training.^16^, ^17^ Conversely, the AIP starts with pre‐defined templates and improves the plan quality iteratively based on the feedback from each round of optimization.^18^ Due to its functioning similarity to manual planning, the AIP has received numerous attention and multiple groups have carried out AIP studies on various platforms.^19^, ^20^ For example, the AutoPlanning module in the Pinnacles (Philips, Fitchburg, WI) treatment planning system (TPS) is a commercial AIP module, validated in different anatomical sites.^21^ Although AIP exhibited many aforementioned features, one of the major barriers to AIP was the planning efficiency limited by the iterations.^22^ Therefore, a critical point to applying AIP in clinical practices was to reduce the necessary iteration while maintaining high plan quality.

Recently, various TPSs have provided application programming interfaces, for example, the Eclipse scripting application programming interfaces (ESAPI) and the Raystation scripting. These programming interfaces provide an avenue to automate various tasks of the treatment planning process in a customizable way.^23^, ^24^ Additionally, multiple pieces of literature have demonstrated that manual errors have been the most frequent cause of failure in risk assessment of the overall radiotherapy treatment process, whereas reducing handoffs between the planner and the TPS is an effective measure to mitigate risks in treatment planning.^25^, ^26^, ^27^

In this work, we focused on developing an AIP‐based treatment planning framework via the ESAPI, tested the plan quality in a LARC patient cohort treated with the SIB‐VMAT technique, and integrated the developed framework into a clinical workflow. To make the AIP framework practical, a particular effort was devoted to reducing the required iterations in AIP while delivering high‐quality treatment plans. To validate the previous claims, the plan quality and planning time comparisons were performed between the automatic plans (AP) and the manual plans (MP) on a large patient dataset.

## 2 MATERIAL AND METHODS

### 2.1 Patient cohort

A cohort of 106 LARC patients previously treated with two‐arc coplanar VMAT in 2021 was retrospectively selected from the clinical database. This retrospective study was ethically approved by the institutional review board, and written informed consent was waived due to the retrospective and observational nature of the study. All patients were treated and simulated with thermoplastic fixation in the head‐first‐supine positions. The Computed Tomography scanning range was defined from the superior border of the second lumbar vertebra to the perineum, with a 5‐mm slice thickness. The target structures included the gross tumor volume (GTV) and the clinical target volume (CTV). Subsequently, a uniform 5‐mm margin was added to the GTV and the CTV, creating the planning gross tumor volume (PGTV) and the planning target volume (PTV), respectively. The considered OARs included the urinary bladder, femoral heads, small bowel, and red marrow. The clinical protocol defined a 25‐fraction regimen with the SIB‐VMAT technique, where 45 Gy (1.8 Gy/fraction) and 50 Gy (2.0 Gy/fraction) were prescribed to the 95% volume of the PTV and PGTV, respectively.^4^, ^7^

### 2.2 Automatic iterative planning

**FIGURE 1:**
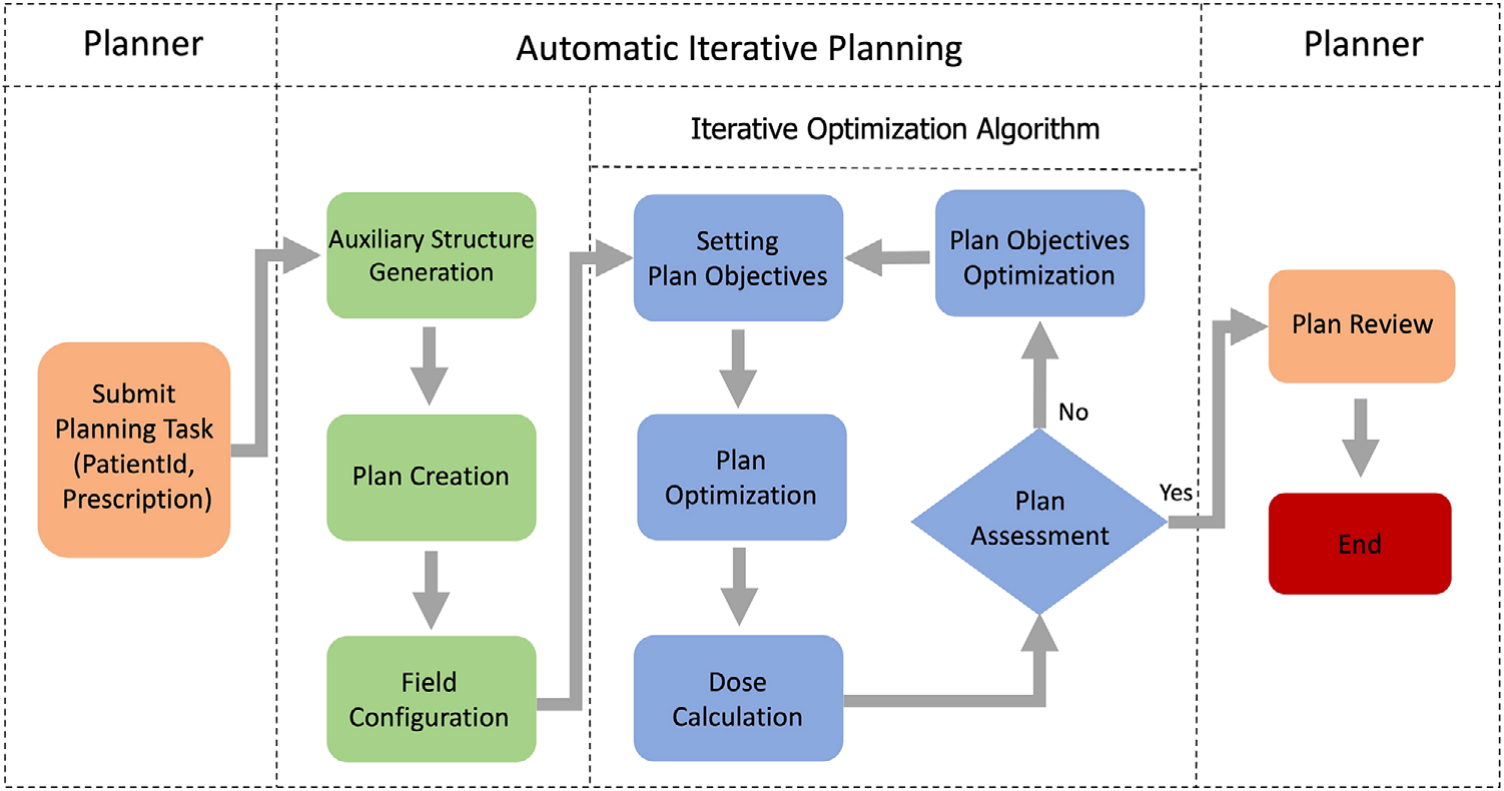
Flowchart of the automatic iterative planning process.

The general automatic planning workflow is depicted in Figure 1. When the contouring of the target and OAR structures were complete, the planner submitted the planning tasks to the automatic planning module via a web‐based interface, providing the patient identifier and corresponding prescriptions. The proposed automatic planning framework proceeded to generate a final plan in several following steps: auxiliary structure generation, plan creation, field configuration, plan optimization, dose calculation, and plan assessment. When the automatic planning process was complete, the final plan was output for manual review. Although the proposed planning method was tested on the LARC patient cohort in this study, the depicted workflow was generic and could be applied to other disease sites. The previously mentioned steps are described separately in this subsection.

#### 2.2.1 Auxiliary structure generation and plan creation

Two auxiliary structures, named IrradVolume and SmallBowel‐Avoid, were generated automatically by the in‐house routines, where the IrradVolume was composed of the volume in the PTV but outside the PGTV with a 10 mm margin, to control the dose fall‐off in the PTV. The SmallBowel‐Avoid was defined as a 100 mm expansion of the PTV in both the anterior and posterior directions within the body structure and with a 5 mm margin away from the PTV. This structure served to encourage fast dose fall‐off near the PTV and to reduce the dose in the abdominal region since the irregular peristaltic motion of the small bowel could introduce significant dose variations.^28^, ^29^

A new plan was generated starting from the plan templates after creating the auxiliary structures. The pre‐configured plan templates were built via the clinical protocol manager in the Eclipse TPS and loaded automatically via the ESAPI, given the input treatment type and prescription. Identical to the clinical practice, the delivery isocenter was placed at the geometric center of the PTV. A coplanar, double‐arc VMAT technique was utilized, comprising a clockwise and a counter‐clockwise arc, with gantry angles 1$81^{\circ }$‐1$79^{\circ }$ and 179
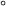
‐181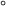, respectively. The photon energy was set at 10 MV. Photon Optimizer^30^ and AcurosXB algorithm^31^ were utilized for the inverse planning and dose calculation respectively, where the voxel size was set at 2.5 mm^3^.

The field configuration module aimed to define the collimator angles and field widths. To ensure a fair comparison between the manual and automatic plans, the AIP adopted the field configuration commonly used by the planners in our clinic. The collimator angles of the two fields were set at 85° and 5°.^32^ The two near‐orthogonal fields aimed to create a concave dose distribution, sparing the urinary bladder while ensuring target coverage. The field width is determined according to the projection boundary of the PTV at all gantry angles.

#### 2.2.2 Iterative optimization algorithm

The Iterative Optimization Algorithm is an iterative method that consists of five main steps at each iteration: setting plan objectives, plan optimization, dose calculation, plan assessment, and plan objectives optimization, as shown in Figure 1. Note that each iteration in automatic planning is equivalent to an entire optimization process in manual planning routine, that is, from objective function setting to optimization convergence. Similar to the manual planning process, the AIP adaptively adjusted the planning objectives per iteration to obtain the final plan. In this work, several types of planning objectives were used to formulate the optimization problem: the minimum/maximum dose, the mean dose, and the DVH objectives (the minimum dose for a specific volume). All these objectives were specified by two adjustable parameters: the dose value and the corresponding weight. To reduce the dimension of the tunable parameters, only one parameter was modified per objective, that is, either the dose or the weight. Consequently, the introduced objectives were divided into two categories: the dose objectives and the weight objectives. The weight objectives included the target‐related objectives, where the corresponding doses were fixed according to the prescriptions. Meanwhile, the dose objectives included the OAR objectives, since the dose values were frequently adjusted to achieve optimal OAR sparing. A set of ideal dose objectives was defined to guide the objective adjustment, as is shown in Table 1, where the ideal objectives of the OARs were all set to 0 to maximize the OAR sparing. An objective‐dose difference (Diff) between the ideal dose objectives (ID) and the currently achieved dose metrics (D) was then introduced:

(1) $$Diff_{i}^{k}=\left(D_{i}^{k}-ID_{i}^{k}\right)\Delta_{i},\;k,i\in Z,$$

(2) $$\Delta_{i}=\left\{\begin{array}{cc} 1 & \mathrm{for}\;\mathrm{lower}\;\mathrm{dose}\;\mathrm{objective}\;\\ -1 & \mathrm{for}\;\mathrm{upper}/\mathrm{mean}\;\mathrm{dose}\;\mathrm{objective} \end{array}\right.\;$$

**TABLE 1:** Plan template of LARC SIB‐VMAT automatic iterative planning, where all the tunable parameters in the AIP module are marked with asterisks. $D_{0\%}$ and $D_{100\%}$ in table represent $D_{max}$ and $D_{min}$ of the respective structures.

Structure	Type	Plan objectives			Ideal objectives		Amplitude conversion factor	Initial adjustment amplitude (Gy)
		Volume (%)	Dose (Gy)	Weight	Volume (%)	Dose (Gy)		
PTV	Lower	100	46	200^*^	95	45	100	–
PGTV	Lower	100	51	200^*^	95	50	100	–
CTV	Lower	100	46	200^*^	100	45	100	–
GTV	Lower	100	51	200^*^	100	50	100	–
body	Upper	0	51	500^*^	0	53	100	–
Irrad volume	Upper	0	47	200^*^	2	48.5	100	–
Urinary bladder	Mean	–	18^*^	200	–	0	–	2
Femoral heads	Mean	–	9^*^	200	–	0	–	2
Bone marrow	Mean	–	18^*^	200	–	0	–	2
Small bowel‐avoid	Mean	–	19^*^	200	–	0	–	2

The Diff encompasses two aspects: assessing whether the dose meets the constraints (relative to the objective type) and quantifying deviation from the ideal dose, where i denotes the objective index, k the current iteration index, and $\Delta_{i}$ the directional parameter for the ith objective. In addition, the ‘lower dose objective’ ensures that the dose delivered to the specified region or point does not fall below the defined minimum threshold. Similarly, the ‘upper dose objective’ and ‘mean dose objective’ ensure that the point/mean dose does not exceed the defined maximum threshold.

At each iteration, the dose‐objective adjustment (ΔDose) and weight‐objective adjustment (ΔWeight) are added to the dose and weight parameters of plan objectives (marked with asterisks in Table 1). The adjustment on the dose and the weight objectives followed two different strategies:

1. For the dose objectives, a pseudo‐gradient $g_{i}^{k}$ was introduced to quantify the effectiveness of the previous adjustment, defined as
(3) $$g_{i}^{k}=\left(Diff_{i}^{k}-Diff_{i}^{k-1}\right)\;/\Delta Dose_{i}^{k-1},$$

where $Diff_{i}^{k}$ and $Diff_{i}^{k-1}$ denote the ith objective distance in the kth and k−1th iterations, and $\Delta Dose_{i}^{k-1}$ the adjustment amplitude of the ith objective in the k−1th iteration. Additionally, $g_{i}^{0}$= 1 and $g_{i}^{k}$= 0 if $\Delta Dose_{i}^{k-1}$= 0. The dose‐objective adjustment (ΔDose) in the current iteration k was subsequently given by

(4) $$\Delta Dose_{i}^{k}=\max\left(0,\tanh\left(2*g_{i}^{k}\right)\right)\;*\Delta Dose_{i}^{k-1}*\Delta_{i},$$

Based on the initial adjustment $\Delta Dose_{i}^{0}$, the kth adjustment takes into account the progress of dose improvement from the k−1th adjustment, that is, the pseudo‐gradient $g_{i}^{k}$. The tanh function serves to limit the maximum tuning amplitude per iteration and adjust the amplitude of the adjustment converged quickly to zero since $\Delta Dose_{i}^{k}$ would be drastically reduced if little improvement were made in the k−1th iteration.

2. The weight‐objective adjustment (ΔWeight) was defined as
(5) $$\Delta Weight_{i}^{k}=\min\left(\max\left(-Diff_{i}^{k}*\eta_{i},-100\right),\;100\right),$$

The amplitude conversion factor ($\eta_{i}$) is a hyperparameter controlling within the algorithm that relates to the weight amplitude adjustment setting, where $\eta_{i}$= 100 indicates that means that a difference of 1 Gy corresponds to an adjustment of weight 100. To improve stability, the min/max function was introduced to limit $\Delta \mathrm{Weigh}t_{i}^{k}$ in the range from ‐100 to 100, and the weight was should be constrained within within [0, ‐1000] to comply within the TPS requirement.

**FIGURE 2:**
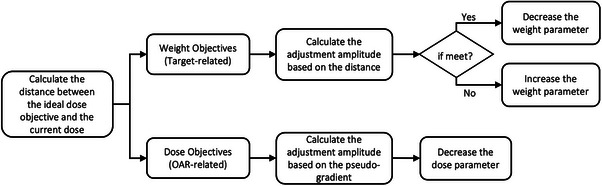
Flowchat of plan objectives optimization strategy.

The purpose of the adjustment is to minimize the distance to the ideal dose objectives, similar to manual planning operations aiming to minimize the dose of OARs while maintaining adequate target coverage. In addition, the distance can be effectively utilized to guide the adjustment for target‐related objectives with clear ideal dose objectives that are aligned with the prescriptions. However, it cannot be applied to OAR‐related objectives because the ideal objectives vary between patients and it is difficult to determine them prior to the actual optimization. Therefore, feedback from previous adjustments on distance changes, (i.e., pseudo‐gradient), is more suitable for tuning guidance. The aforementioned parameter adjustment strategy is summarized in Figure 2. Finally, two stopping criteria were introduced for the optimization iteration: 1. A maximum iteration number of 15 was used; 2. Minimum adjustment amplitudes for the dose and weight objectives were set at 0.1 Gy and 20, respectively^a^ Subsequently, final dose calculation and plan normalization were performed to meet the prescription requirements.

### 2.3 Implementation

**Planning template**: The iterative optimization started with a pre‐defined template that included initial plan objectives, ideal dose objectives, and initial adjustment steps, as shown in Table 1. The ideal objectives for the target structures in the template were defined according to clinical protocols. In this studied LARC cohort, the institutional SIB prescription specified 95% coverage of the PTV with 45 Gy, 95% coverage of the PGTV with 50 Gy, and the maximum dose in the patient less than 110% of the highest prescription. The initial plan objectives for the OARs were set empirically based on the average dose metrics among historical plans or planners’ experiences. In addition, two fixed objectives were also included in the template: the normal tissue objective with a fixed weight of 600 to control the dose spillage, and the monitor unit (MU) objective where the minimum MU and the maximum MU were set at 600 and 640, respectively. To maintain a reasonable level of complexity during the optimization, an MU penalizing weight of 80 is employed.

**FIGURE 3:**
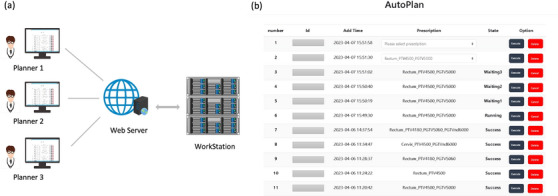
The web‐based automatic planning system, where (a) shows the data interaction mode between the planners and the workstation, and (b) displays the graphical user interface of the automatic planning web page, where the masked column denotes the patient identifier.

**Clinical deployment**: To facilitate the usage of the proposed auto‐planning approach in clinical practice, a web‐based service was implemented for user interfacing and task scheduling, built upon the Django package.^33^ The main workflow is shown in Figure 3(a) and (b), where the planners submitted the planning tasks via a webpage, providing the patient's ID and corresponding prescriptions. The web service also provided real‐time monitoring of the planning process, so that the planner could review or fine‐tune the final plan once the submitted planning task was complete. The current task scheduling system performed each auto‐planning task in a sequential manner, where the requested patient data were retrieved from the clinical database via the ESAPI. The generated data during the planning process was saved in an independent MySQL database for post‐planning analysis. In terms of hardware, the web service and the AIP procedure were both hosted on a workstation with an Intel Xeon Gold 6132 CPU and two NVIDIA Tesla P100 GPUs. On the same system, an Eclipse TPS v15.6 (Varian, Palo Alto, CA) was installed to perform the inverse planning tasks.

### 2.4 Plan evaluation

To compare the performance of our AIP method, the plan quality of the APs was compared with the MPs in the aforementioned cohort with 106 LARC patients. In addition, the MPs were separated into 3 groups based on the planners’ experience: junior, intermediate, and senior, whose corresponding planning experiences were < 5 years, 5–10 years, and > 10 years, respectively. All 106 cases were allocated to these three groups, with 26 cases in the junior group, 38 cases in the intermediate group, and 42 cases in the senior group. A total of 6 planners were included, with each group including 2 planners. To perform a fair comparison between the MP and the AP, the AIP aligned with the planning strategies of the planners, using identical dose prescriptions for the target volumes, and the dosimetric goals for the OARs were specified following the “as low as reasonably achievable” (ALARA) principle. The group‐by‐group comparison in plan quality allowed us to assess the effectiveness of our AIP solution across different levels of planning experience.

The plan quality was assessed by critical DVH metrics, MU metrics and complexity metrics.^34^ For the PTV, the max dose ($D_{\max}$), $D_{95\%}$, $D_{98\%}$ and the conformity index (CI) were compared, while $D_{2\%}$ $D_{95\%}$, $D_{98\%}$, and the homogeneity index (HI) were used for the PGTV. According to vantRiet et al.^35^ and Wu et al.,^36^ the CI and the HI were defined as: $\mathrm{CI}\;=\left(V_{T,\mathrm{ref}}/V_{T}\right)\times \left(V_{T,\mathrm{ref}}/V_{\mathrm{ref}}\right)$, where $V_{T,\mathrm{ref}}$ refers to the volume of the target receiving a dose equal to or greater than the reference dose, $V_{T}$ the volume of the target, $V_{\mathrm{ref}}$ the volume receiving a dose equal to or greater than the reference dose. $\mathrm{HI}\;=|D_{2}-D_{98}|\;/D_{p}$, where $D_{p}$ refers to the prescription dose, $D_{x\%}$ the dose received by x% of the volume. Referring to the compliance criteria in RTOG 0822 protocol, the mean dose ($D_{\mathrm{mean}}$), the max dose (D_max_), $V_{40\;\mathrm{Gy}}$ and $V_{45\;\mathrm{Gy}}$ were compared for the urinary bladder and the femoral heads.^37^ For the small bowel, $D_{2\;\mathrm{cc}}$, $V_{45\;\mathrm{Gy}}$, $V_{40\;\mathrm{Gy}}$ and $V_{35\;\mathrm{Gy}}$ were used while $D_{\mathrm{mean}}$, $V_{40\;\mathrm{Gy}}$, $V_{20\;\mathrm{Gy}}$ and $V_{10\;\mathrm{Gy}}$ for the bone marrow^38^, ^39^ and $D_{\mathrm{mean}}$ and $D_{\max}$ were evaluated for the SmallBowel‐Avoid. Wilcoxon signed‐rank tests (p<0.05 considered statistically significant) were used to evaluate the statistical significance between AP and MP.

### 2.5 Plan quality assurance

Based on the differences of planning complexity between MPs and APs, we divided a total of 108 cases into three groups evenly. From each group, 10 cases were selected randomly, resulting in a subset of 30 cases for plan quality analysis and blind evaluation. Plan quality assurance was performed using the Portal Dosimetry module (Varian Medical Systems, Palo Alto, CA USA). Dosimetric data were evaluated using the gamma analysis method, with acceptance criteria set at a dose difference of 3%, a distance of 2 mm, and a threshold value of 10%. To determine the agreement between pairs of fluence maps, the criteria of γ[3%,2 mm] < 1 needed to be met in 95% of the analyzed field points. In addition, a blind evaluation was conducted by an experienced physicist to compare the APs and MPs.

## 3 RESULTS

With the proposed AIP system, 106 APs were successfully generated without any manual intervention, within a span of 50 hours totally. On average, the automatic planning time was 36.8 ± 6.5 minutes, where 34.8 ± 6.5 minutes were spent on the iterative optimization process. For manual planning, the time cost was approximately 40 min in our clinical center. The average iteration number of plan optimization was 6.8 ± 1.1.

**FIGURE 4:**
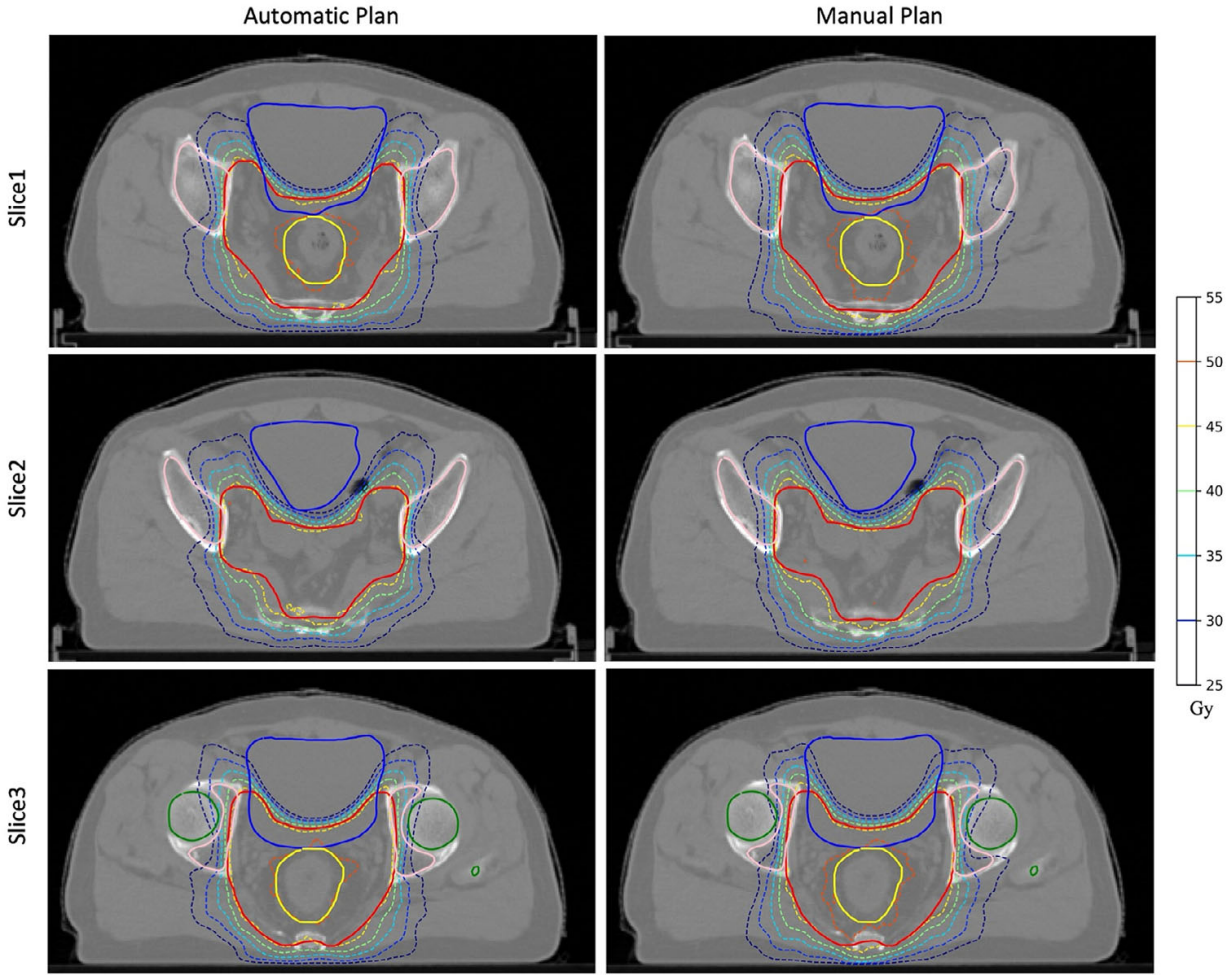
Isodose distribution of 3 different slices from a sample patient plan. The solid lines represent the structures with the structures of PGTV (yellow), PTV (red), urinary bladder (blue), femoral heads (green), and bone marrow(pink) while the dashed lines represent the isodose line.

All automatic plans were demonstrated to meet clinical requirements through comparison and evaluation of DVH metrics and planar doses between MP and AP. Table 2 provides a quantitative comparison between MP and AP in terms of target coverage, dose conformity, dose homogeneity, and OAR sparing. All MP and AP successfully achieved the clinical prescription requirements in terms of target coverage. When comparing the homogeneity and conformity of dose distribution in the target volume using HI/CI analysis, both AP and MP yielded similar outcomes. However, AP exhibited significantly better performance (p < 0.001) in controlling hot spots, (specifically, $D_{\max}$ in PTV and $D_{2\%}$ in PGTV), while exhibiting lower $D_{98\%}$ in the target volume (p < 0.001). For the MU metrics and complexity metrics, AP were significantly higher than MP (p<0.001), but with a small absolute difference. The mean MU of MP was 620.72 while the mean MU of AP was 644.92, and the mean complexity of the MP and AP were 0.122 and 0.126, respectively. In the OARs, AP had shown significantly better sparing than MP in the femoral heads, the bone marrow, and the SmallBowel‐Avoid. Specifically, the mean dose reductions in these three structures were 0.53 Gy, 1.18 Gy, and 1.00 Gy, respectively (p<0.001). AP and MP had comparable dose sparing in the urinary bladder, as measured by the $D_{\mathrm{mean}}$, $D_{\max}$, $V_{45\;\mathrm{Gy}}$ and $V_{40\;\mathrm{Gy}}$. Regarding the bone marrow and the small bowel, AP showed a significant but slight reduction. Figure 4 demonstrates the isodose distribution of three different slices from a sample patient plan. The isodose curve of the AP exhibited a higher degree of overlap with the target volume compared to MP and the AP provided improved protection of the bone marrow. However, a minor volume with lower dose was observed at the target volume boundary in AP. Additionally, a plan quality assurance was conducted on the 30 cases. The gamma pass rate for the MPs was slightly higher than that for the APs (P < 0.05). However, both the MPs and APs passed the gamma analysis where the average gamma pass rate for the MPs was 99.46 ± 0.43 and the average gamma pass rate for the APs was 99.12 ± 0.49. Overall, AP were comparable to MP in plan quality and there is a different tradeoff between them that AP had better performances in dose conformity, hot spot controlling, and OAR sparing but with higher plan complexity and lower $D_{98\%}$ in the target volume.

**TABLE 2:** Dosimetric metric statistics of manual plans and automatic plans for LARC SIB‐VMAT. Two groups of plans are compared using the Wilcoxon signed‐rank test (*p* < 0.05), with significant terms marked in bold.

	Metrics	Manual plans	Automatic plans	*p*‐value
PTV	$D_{\max}$ (Gy)	54.00 (±0.61)	53.61 (±0.33)	**<0.001**
	$D_{98\%}$ (Gy)	44.73 (±0.42)	44.28 (±0.12)	**<0.001**
	$D_{95\%}$ (Gy)	45.43 (±0.30)	45.07 (±0.09)	**<0.001**
	CI	0.90 (±0.02)	0.91 (±0.01)	**<0.001**
PGTV	$D_{2\%}$ (Gy)	53.08 (±0.51)	52.48 (±0.20)	**<0.001**
	$D_{98\%}$ (Gy)	50.32 (±0.33)	49.84 (±0.13)	**<0.001**
	$D_{95\%}$ (Gy)	50.67 (±0.28)	50.22 (±0.12)	**<0.001**
	HI	0.060 (±0.011)	0.059 (±0.004)	0.03
Urinary bladder	$D_{\mathrm{mean}}$ (Gy)	18.71 (±3.08)	18.36 (±3.25)	0.22
	$D_{\max}$ (Gy)	50.89 (±1.58)	50.80 (±1.25)	0.40
	$V_{45\;\mathrm{Gy}}$ (%)	11.60 (±5.54)	11.18 (±5.30)	**0.002**
	$V_{40\;\mathrm{Gy}}$ (%)	17.00 (±6.45)	16.37 (±6.43)	**<0.001**
Femoral heads	$D_{\mathrm{mean}}$ (Gy)	8.64 (±0.88)	8.11 (±1.27)	**<0.001**
	$D_{\max}$ (Gy)	36.60 (±3.25)	34.81 (±3.43)	**<0.001**
Bone marrow	$D_{\mathrm{mean}}$ (Gy)	19.98 (±2.41)	18.80 (±2.20)	**<0.001**
	$V_{40\;\mathrm{Gy}}$ (%)	5.92 (±2.07)	4.24 (±1.57)	**<0.001**
	$V_{20\;\mathrm{Gy}}$ (%)	47.08 (±9.51)	41.93 (±6.83)	**<0.001**
	$V_{10\;\mathrm{Gy}}$ (%)	79.82 (±11.31)	78.22 (±11.75)	**<0.001**
Small bowel	$D_{2\;\mathrm{cc}}$(Gy)	47.04 (±6.56)	46.80 (±6.49)	**<0.001**
	$V_{45\;\mathrm{Gy}}$ (cc)	27.61 (±26.09)	25.85 (±24.90)	**<0.001**
	$V_{40\;\mathrm{Gy}}$ (cc)	45.88 (±37.64)	43.16 (±36.14)	**<0.001**
	$V_{35\;\mathrm{Gy}}$ (cc)	65.08 (±48.52)	59.48 (±45.46)	**<0.001**
SmallBowel‐Avoid	$D_{\mathrm{mean}}$ (Gy)	16.90 (±2.52)	15.90 (±2.12)	**<0.001**
	$D_{\max}$ (Gy)	45.70 (±1.52)	45.18 (±1.68)	**0.01**
MU	–	618.8 (±39.6)	644.9 (±3.0)	**<0.001**
Complexity	–	0.122 (±0.011)	0.126 (±0.007)	**<0.001**

A blind evaluation was performed for 30 cases to select the superior plans based on a comprehensive assessment of plan metrics such as dose distribution and DVH metrics. An experienced physicist confirmed these dosimetric findings and assessed that APs were better or equal to MPs in 23 cases. In the remaining 7 cases, MPs achieved lower bladder doses, while APs demonstrated superiority in the dose fall‐off and maximum dose within the PTV. The evaluator considered bladder dose prioritization as more critical, which is also a potential direction for adjustment of automatic planning.

**FIGURE 5:**
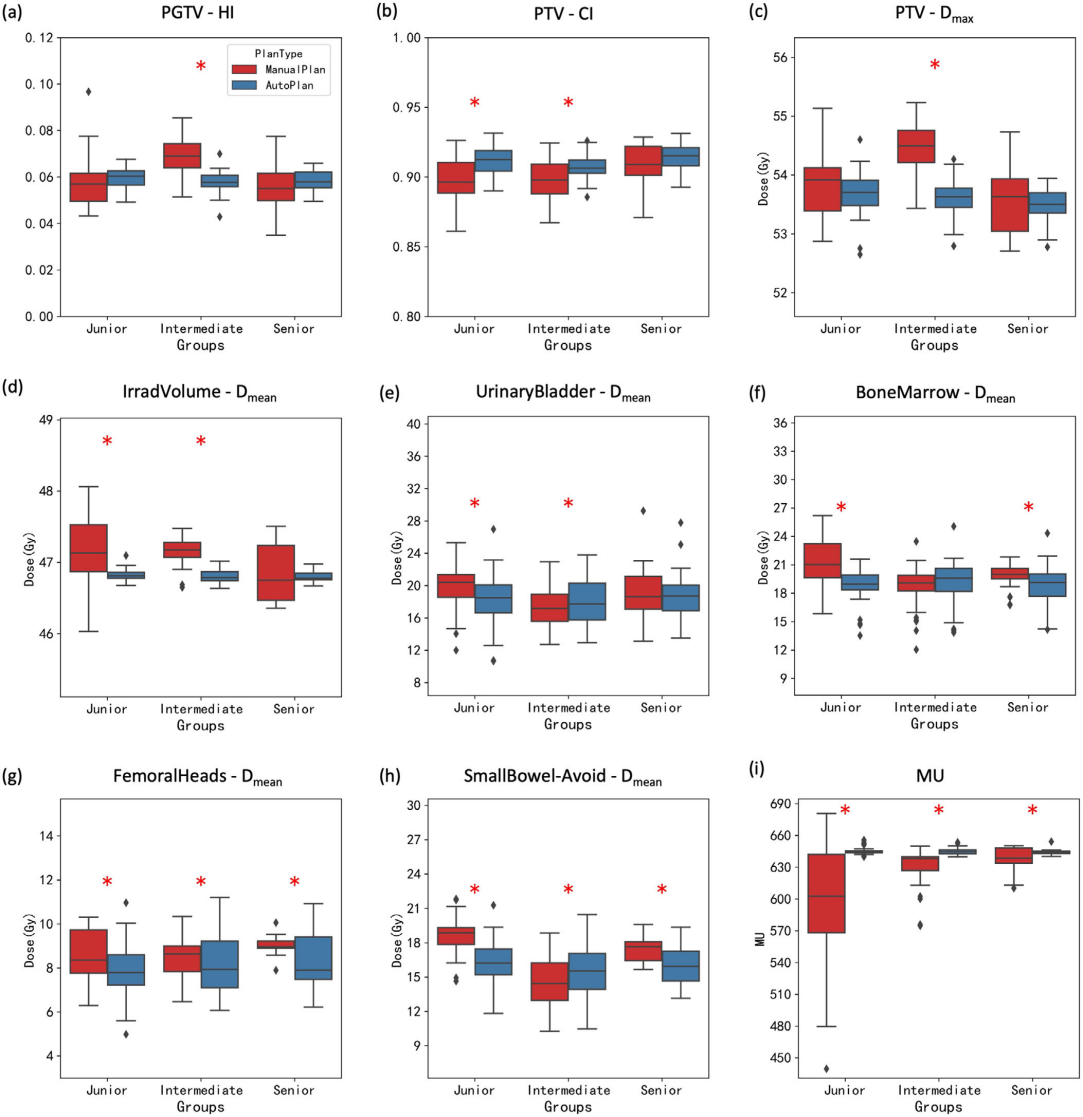
Dosimetric indicator comparison between MP and AP in different groups of planners. Red asterisks indicate statistical significance.

The plan evaluation was conducted across 3 groups of planners with variable experiences and the comparison results were summarized in Figure 5. In the junior group, the comparison indicated that AP exhibited significant improvements compared to MP on almost all dosimetric indicators, except for the HI in the PGTV. In the intermediate group, AP had significant improvements in dose conformity, dose homogeneity, and dose fall‐off within the target volume. In terms of dose sparing, MP resulted in a lower mean dose to the urinary bladder, while AP resulted in a lower mean dose to the femoral heads, highlighting different preferences between the planners and the AIP approach. In the senior group, comparing the dose distribution in the PTV, similar DVH metrics were observed between the planners and the AIP approach. However, AP had a better dose sparing in the femoral heads and the bone marrow than MP. As is shown in Figure 5(i), there was a significant difference in the MU values between AP and MP across all three groups, with a larger difference observed in the junior group. In addition, the error bars in Figure 5 illustrated that the plan consistency of AP was superior to that of MP in all three groups.

**FIGURE 6:**
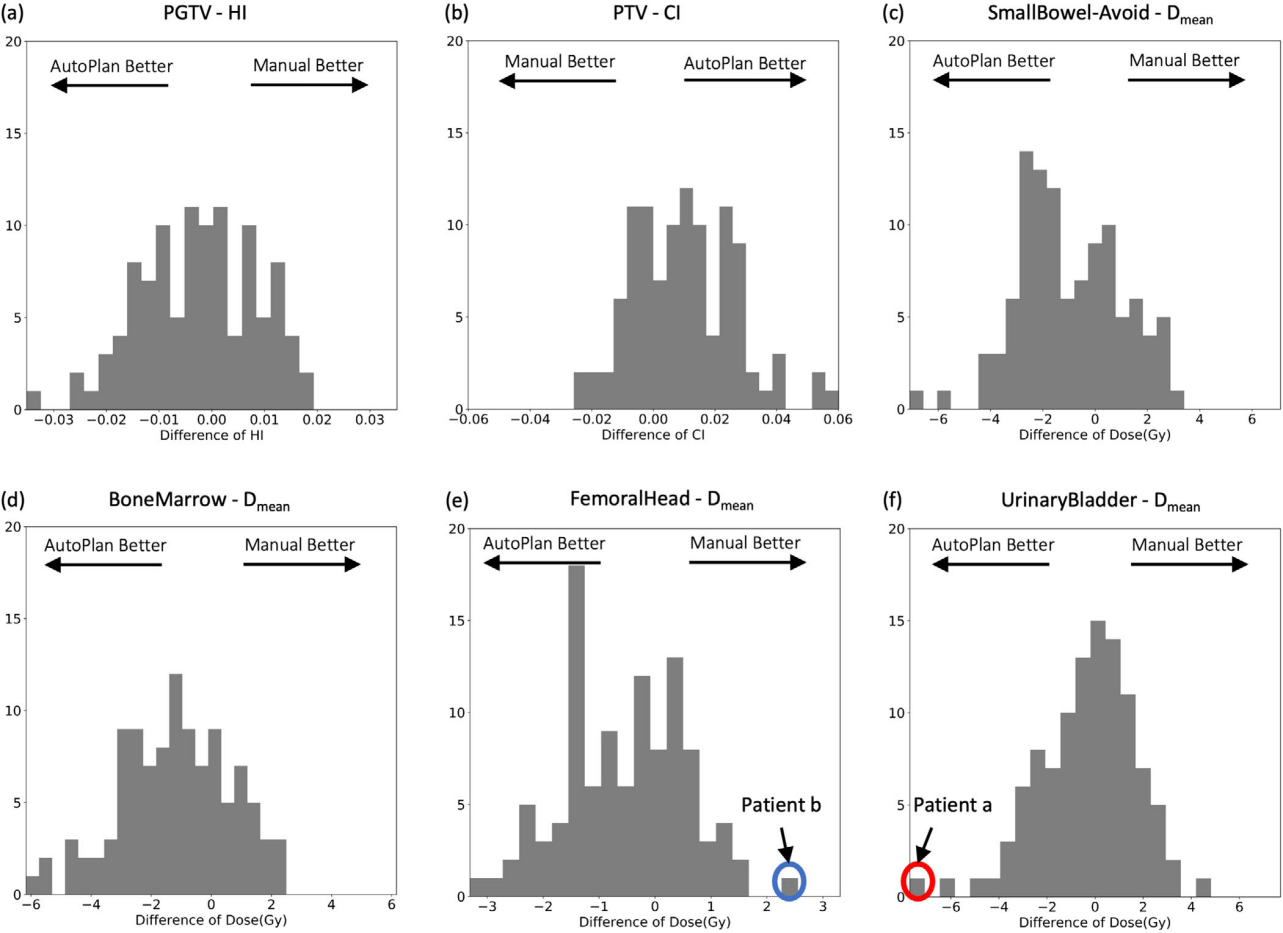
Histograms of difference between APs and MPs for 6 dosimetric indicators. Two outliers were chosen as typical cases where APs achieved better or worse metrics than MPs highlighted by the red and blue circles, respectively.

**FIGURE 7:**
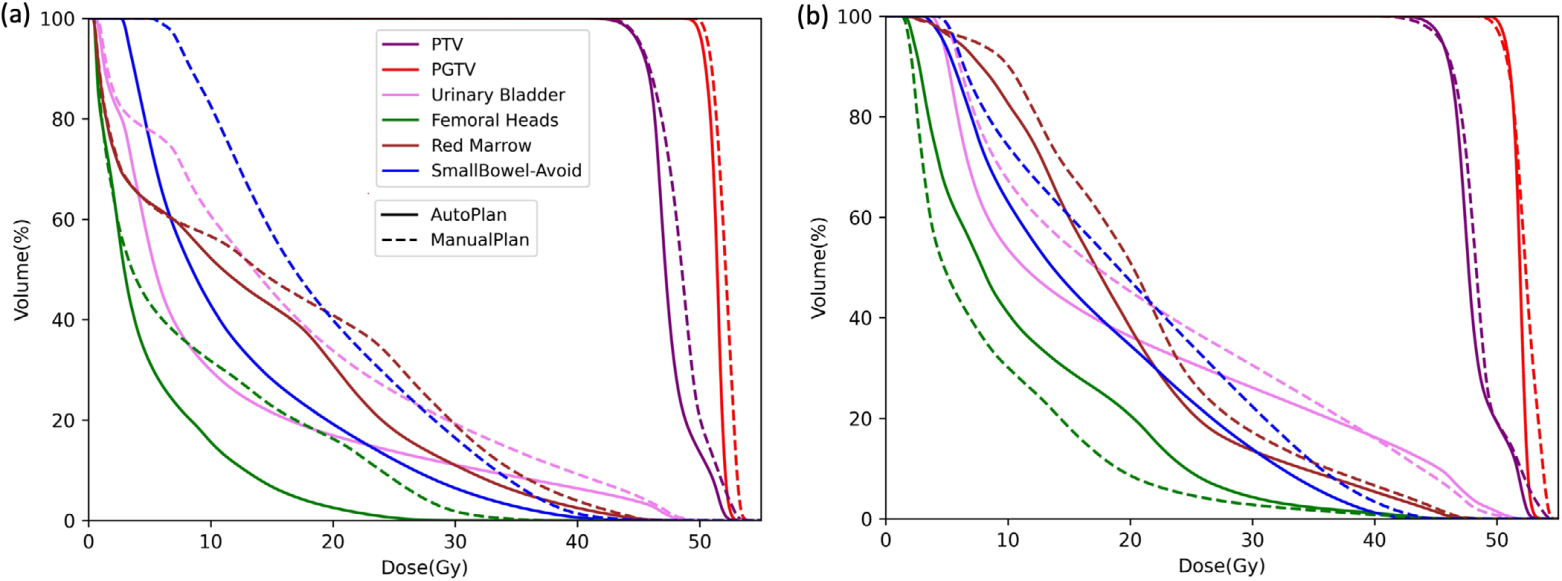
Detailed DVH comparisons between AP and MP for two outlier instances. The DVH of patient a in Figure 6 plotted in (a) shows that the urinary bladder *D*_mean_ of the AP is extremely lower than that of the MP; the DVH of patient b in Figure 6 plotted in (b) shows that the femoral head *D*_mean_ of the MP is significantly lower than that of AP.

Considering the diversity of patients, it is crucial to determine the appropriate scope of AIP application to ensure patient safety. In this regard, comparisons between the AP and MP were conducted for each patient, and the detailed distribution of the differences (AP—MP) was presented in Figure 6, including the HI, the CI, and the mean dose of several OAR structures. The overall difference distribution indicated that AP outperformed MP for the majority of the cohort on the evaluated metrics but with several visible outliers. Two outlier cases with extreme DVH metric differences between the two plans were selected for further analysis, highlighted by the red and blue circles in Figure 6, respectively. The DVH curves of AP and MP were plotted and compared for both cases in Figure 7. In Figure 7(a), the MP is suboptimal in every aspect compared to the AP and it is crucial to emphasize that the improvement in AP comes without compromise. In Figure 7(b), the difference between the two plans was caused by different trade‐offs between the urinary bladder and the femoral heads, where both plans were considered clinically acceptable.

## 4 DISCUSSION

In this study, an AIP system was developed and evaluated for its performance versus MPs in LARC SIB treatment. We provided detailed assessments on a cohort of 106 patients and proposed implementation methods for integrating AIP into the clinical pipeline.

In our AIP approach, the interactive optimization process is effective and efficient for inverse planning due to its ability to rapidly converge using pseudo‐gradient information. Compared to other optimization strategies, such as tree‐based exploration,^20^ Bayesian optimization,^22^ and particle swarm optimization,^40^ our algorithm reduced the required iterations in the optimization process within a clinically acceptable range. The proposed iterative approach aims to solve the plan quality improvement part in the auto‐planning process. Unlike data‐driven methods, the effectiveness of our AIP approach relied on a general strategy of iterative improvement, which allowed for flexible adaptation to modifications in clinical requirements. Thus, many historical plans can be utilized for validation rather than training models, where the current performed studies included patients with varying target volumes (922 ± 154 cm^3^) and urinary bladder volumes (311 ± 118 cm^3^), indicating a certain level of robustness of the proposed approach. To showcase the optimization process of our algorithm, an example iteration process is presented in Table S3 of the supplementary material, including the changes in parameters and the corresponding adjustments made during each iteration. Furthermore, Figure S3 in the supplementary material presents the DVHs of the AP in the initial three iterations and the final iteration throughout the optimization process, illustrating a continuous improvement in plan quality as the iterative optimization progresses.

In our implementation, the automatic planning system focuses on setting mean‐dose objectives for the OARs in optimization based on the planners' experience. And the automatic planning supports the inclusion of other types of constraints in optimization, which can be found in the case study of NPCs in the supporting materials. In addition, the initial dose parameters of OAR objectives provide a mean starting point based on previous plans of similar prescription and tumor sites. To evaluate the planning sensitivity of initial dose parameters, 30 additional plans were generated with more generic initial dose objectives (shown in Table S1) and the detailed experimental setups and dosimetric comparison (shown in Figure S1) can be found in the supplementary materials. The comparison with the MPs has demonstrated that initial dose parameters did not significantly impact the final plan quality. However, the iterative planning time with the objectives based on historical plans was significantly shorter (36.8 ± 6.5 minutes) compared with that with more general initial dose parameters (58.8 ± 12.2 minutes). Additionally, it is important to note that different initial weight parameters (shown in Table S2) influenced the plan quality more significantly compared to the dose objectives and the initial weight parameters affect the trade‐offs between objectives. An additional experiment were performed by varying the initial objective weights and observing the final plan variation. The resulting DVHs shown in Figure S2 of the supplementary materials indicate that increasing the weight parameter can lead to better achievement of the corresponding objective, but this improvement comes at the expense of other conflicting objectives. A possible explanation is that changes in weight parameters induced more drastic variations in the loss values with the current functional form reflecting the objectives. Consequently, the initial weight parameters can be adjusted to align with the preferences of different planners for customization.

RTOG 0822 protocol has provided compliance criteria for patients diagnosed with LARC, and the DVH metrics of both AP and MP generally met the criteria, except for the Dmax of the urinary bladder. When the urinary bladder overlap with the PGTV, the maximum dose may exceed 50 Gy to maintain high dose coverage in the PGTV. Moreover, the AP lowered the mean dose of the femoral head compared to the MP, but the dose difference of 0.5 Gy may not induce different clinical outcomes. Regarding the bone marrow, the AP demonstrated a significant reduction in the mean dose compared to the MP, with a difference of 1.18 Gy. Studies have shown that minimizing the mean dose to the bone marrow can help reduce the risk of hematologic toxicity.^38^ For instance, a mean dose below 22.5 Gy is associated with a 5% risk, and a mean dose below 25 Gy is associated with a 10% risk. The 1.18 Gy reduction achieved by the AP could potentially lower the risk of hematologic toxicity, although further research is needed to quantify the clinical benefits of this dose reduction for specific OARs. Following the ALARA principle, the proposed approach enables lower OAR doses and may benefit the patient.

Plan variation between the planners in different groups was observed in Figure 5, indicating that manual planning is highly dependent on the planner's experiences and individual preferences. In contrast, the AIP approach achieved better quality consistency across a range of patients, generally, yielding similar AP for patients with similar anatomies. Furthermore, the stratification based on the planner's experience demonstrated that planners with less experience might lack attention to MU values or OAR sparing, and consequently produce suboptimal plans, as shown in Figure 5 and Figure 7(a). These results align with the statistical differences between AP and MP presented in Table 2, where the findings indicate that suboptimal plans created by less‐experienced physicists created by less‐experienced physicists lowers the overall quality of manual plans significantly. As previously discussed by Chen et al.,^41^ achieving high‐quality plans requires sufficient time and attention, which can be challenging in clinics with high patient throughput. The proposed AIP approach was able to generate high‐quality plans consistently, thereby overcoming this challenge. These results indicated that the AIP has the potential to replace manual planning. Although some commercial software offered mature automatic planning modules, such as RapidPlan and Autoplanning, our AIP approach focused on providing fully automatic solutions for the entire treatment planning process, rather than just the plan optimization.

It is worth noting that the AIP approach can consistently achieve similar trade‐offs with increasing conflicting objectives, as is the case for more complex treatment sites, e.g. the head‐and‐neck cases. A preliminary experiment was performed to apply the proposed auto‐planning method on 16 treated patients diagnosed with nasopharyngeal carcinoma, and the mean DVH comparison was performed between the MP and the AP for quality evaluation. The comparison revealed that most OARs benefitted better sparing in the AP, except for a single OAR structure (thyroid gland). More importantly, the AP exhibited more consistent DVH metrics compared to the MP, due to identical trade‐off preferences for all the patients. Although structural heterogeneity across patients contributes dominantly to the variance of the DVH metrics, results shown in this work provided evidence of the plan quality consistency improvement with the AIP approach. The detailed experimental setup (shown in Table S4) and results for the NPC patients (shown in Figure S4 and Table S5) were moved into the supplementary materials to keep the manuscript concise.

Integrating the AIP into clinical practice can be a complex and labor‐intensive process. However, the introduction of ESAPI has significantly simplified this process by providing programming interfaces for patient data access and plan modification, which eliminated the need for extensive data configuration and validation, such as patient simulation, beam modeling, and dose calculation engine validation. Consequently, the proposed approach has been demonstrated easily integrated and utilized in the clinical workflow. In addition, web‐based deployment, with its distributed architecture, also provides several advantages for clinical applications. On one hand, it simplifies updates and maintenance of the application for developers, as server‐side changes can be made without requiring client‐side updates. On the other hand, web‐based architecture enhances application efficiency and accessibility for planners.^42^

Interestingly, a former study by Song et al.^19^ investigated the feasibility of automatic planning for rectal cancer on the Pinnacle TPS. Compared to the sork of Song et al., our work differs in the following 3 aspects: 1) We have proposed a pseudo‐gradient‐based objective adjustment method, whose efficacy has been proved by the resulting plan quality; 2) Our research further analyzed the robustness of automatic planning using a larger dataset and specifically focused on the improvement of plan consistency through automatic planning; 3) Our web‐based application model successfully demonstrated to seamlessly integrate the automatic planning into clinical workflows, with the potential to effectively promote its clinical application. In addition to the previously discussed features, this study may contribute to future works in the following directions: 1) Plan datasets for training significantly influence the performance of learning‐based applications, but they can be challenging to collect due to planner preferences and various clinical scenarios.^16^, ^43^ The AIP can provide a large consistent dataset for learning‐based applications, which may improve the predictive accuracy and potentially lead to better treatment outcomes for patients. 2) The AIP can also serve as a radiation planning assistant for institutions with limited resources where experienced planners are rare, which will also improve the consistency of treatment between institutions and ensure that patients in these areas receive high‐quality care.^44^

One limitation of this work is the planning time due to the interaction mode of ESAPI. Unlike manual planning, objective parameters in the AIP approach can only be adjusted after the plan optimization reaches convergence. To address this issue, exploring methods for an asynchronous adjustment scheme or improving the planning optimization speed would significantly accelerate the planning process. Recently, Men et al.^45^ proposed a novel aperture‐based algorithm for VMAT plan optimization, which takes only 18–31 seconds. This indicated that the planning time in our AIP may still be reduced. Another limitation is the applicability of AIP. Although the AIP can generate excellent SIB‐VMAT plans for most patients, it should be noted that the AIP may not be appropriate for some patients with special anatomies and clinical requirements. This indicates that patient anatomical features should be included in clinical goal formulation. Therefore, future research and ongoing observation are necessary for personalized design.

## 5 CONCLUSION

In this work, an AIP system has been successfully implemented and integrated into the clinical pipeline for generating SIB‐VMAT plans for LARC patients. An iterative optimization algorithm was specifically designed for plan optimization, thereby improving the efficiency of the planning process. Comparisons with large planning datasets have demonstrated that the AIP approach exhibits better planning consistency while maintaining comparable planning quality to manual planning. This indicates that the AIP system can provide reliable and consistent plans, offering an alternative to manual planning processes. By replacing manual planning, the AIP system reduces the burden on clinicians and streamlines their workflow, ultimately enhancing the overall efficiency of clinical work.

## AUTHOR CONTRIBUTIONS

J.L. and R.W. implemented the automated treatment planning framework, drafted the initial manuscript together. Q.W. participated in the framework implementation. K.Y., M.W., and H.Y. performed plan quality evaluation and compared the automated plans with the manual plans. RW, YD and HW designed the study protocol, revised the manuscript, and acquired funding support.

## ACKNOWLEGMENTS

This work was supported in part by National Key Research and Development Project (No. 2019YFF01014405), Science Foundation of Peking University Cancer Hosptial (ZY202410), the Beijing Natural Science Foundation (No. 1212011), National Natural Science Foundation of China (No. 12375335, 12005007).

## CONFLICT OF INTEREST STATEMENT

The author declares no conflict of interest.

## Supporting information



Supporting Information

## Footnotes

a When all the calculated adjustment amplitudes fell below the defined limits, the interactive optimization process was stopped.
