# Supplementary material for: Replacing manual planning with automatic iterative planning for locally advanced rectal cancer VMAT treatment
Source: J Appl Clin Med Phys. 2024 Oct 15;26(1):e14552. doi: 10.1002/acm2.14552 (PMC11713272; doi:10.1002/acm2.14552)
Supplement: Supplementary file 1 — Supporting Information [file ACM2-26-e14552-s001.pdf]

Supporting Materials for "Replacing Manual Planning with Automatic Iterative Planning for Locally Advanced Rectal Cancer VMAT Treatment"

1. The sensitivity of the automatic planning on the initial planning parameters

The initial planning parameter template is a critical component of automated planning as it significantly influences both the efficiency and quality of the planning process. While the template allows for the effective configuration of initial target parameters based on the prescription and criteria outlined in RTOG 0822, determining appropriate initial parameter settings for OAR poses challenges and exhibits significant deviations across different institutions. To address this issue, supplementary experiments will be conducted below to guide the initial parameter setup and assess the impact of different OAR parameters on the automatic planning. These experiments aim to provide recommendations for configuring initial parameters, ultimately enhancing the effectiveness and reliability of the planning process.

1.1 Dose parameters of OARs

In our implementation, the initial dose parameters are determined based on the average values from previous plans with similar prescriptions and tumor sites, as indicated in Table 1. This implementation serves as a reference point for comparison and an additional experiment named "DoseParaTest" was conducted. As shown in Table S1, the dose parameters of OAR were set with wider ranges roughly based on the distance to the PTV. All other parameters were kept consistent with the Reference configuration. Subsequently, a comparative analysis of the plan quality and planning time between the Reference and DoseParaTest was performed on 30 cases.

Using the proposed AIP system, 30 addition plans of DoseParaTest were successfully generated, with an average planning time of 58.8 ± 12.2 minutes. When comparing the plan quality, minor differences in the DVH were observed between the OAR and the target volumes for both the automatic plans of the Reference and DoseParaTest, as depicted in Figure S1. In summary, while the initial dose parameters derived from prior plans had a limited impact on plan quality, setting reasonable initial objectives significantly reduced the time required for planning optimization. Our implementation achieved an average planning time reduction of 22 minutes.

Table S1 Different initial dose parameters setup of OARs

OAR	Type	Dose	
		Reference	DoseParaTest
urinary bladder	Mean	18	25
femoral heads	Mean	9	15
bone marrow	Mean	18	20
SmallBowel-Avoid	Mean	19	25

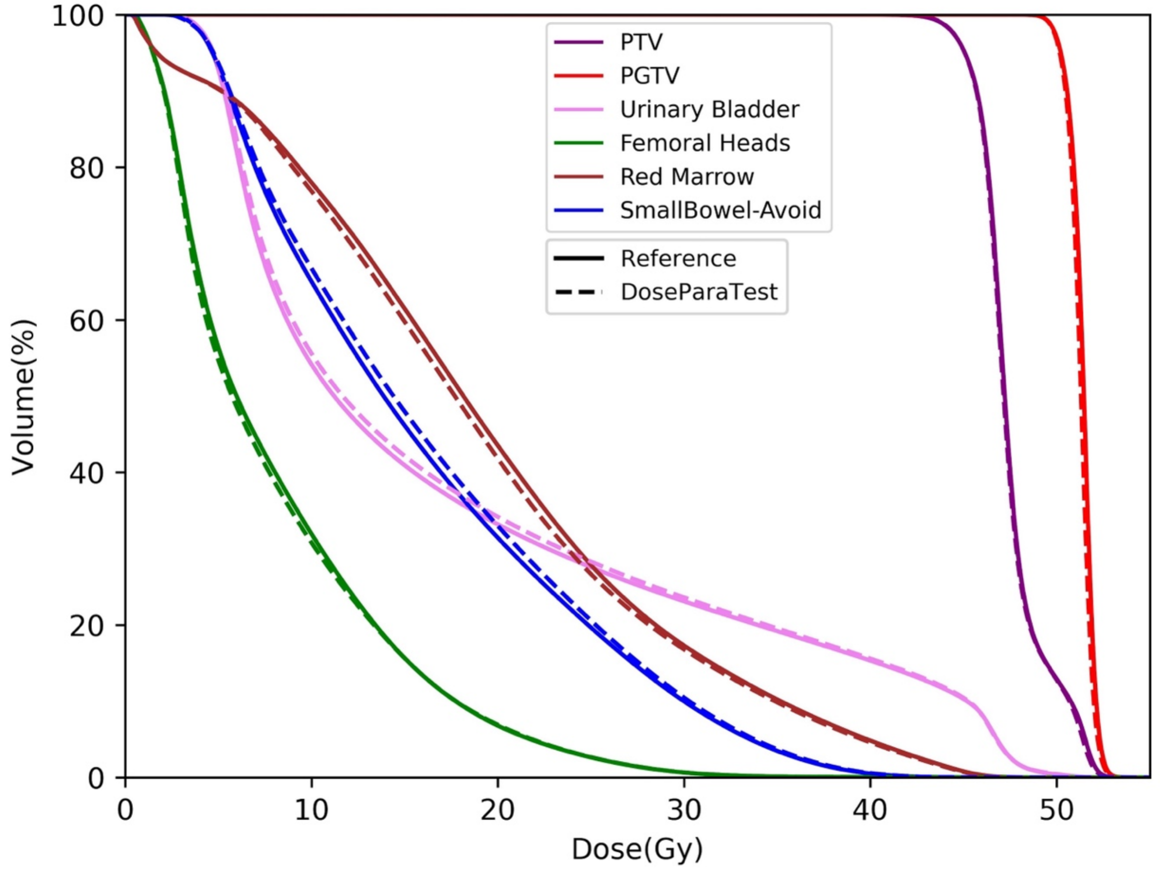

Figure 1 Average DVH comparisons between the Reference plans and the DoseParaTest plans with different initial dose parameters

1.2 Weight parameters of OARs

Weight parameters play a crucial role in determining the importance of optimization goals in the planning process. In our implementation, we conducted three additional experiments to investigate the influence of initial weight parameters on plan quality. Initially, all weight parameters for the OAR were set to 200, and the resulting automatic plans were considered as the reference. The three additional experiments were named WeightTest1, WeightTest2, and WeightTest3, with varying initial weight parameter settings, as indicated in Table S2. Subsequently, a comparative analysis of the plan quality and planning time was performed across the different experimental groups using a total of 30 cases.

Table S2 Different initial weight parameters setup of OARs

OAR	Type	Weight			
		Reference	WeightTest1	WeightTest2	WeightTest3
urinary bladder	Mean	200	250	200	300
femoral heads	Mean	200	200	250	200
bone marrow	Mean	200	200	200	200
SmallBowel-Avoid	Mean	200	200	200	200

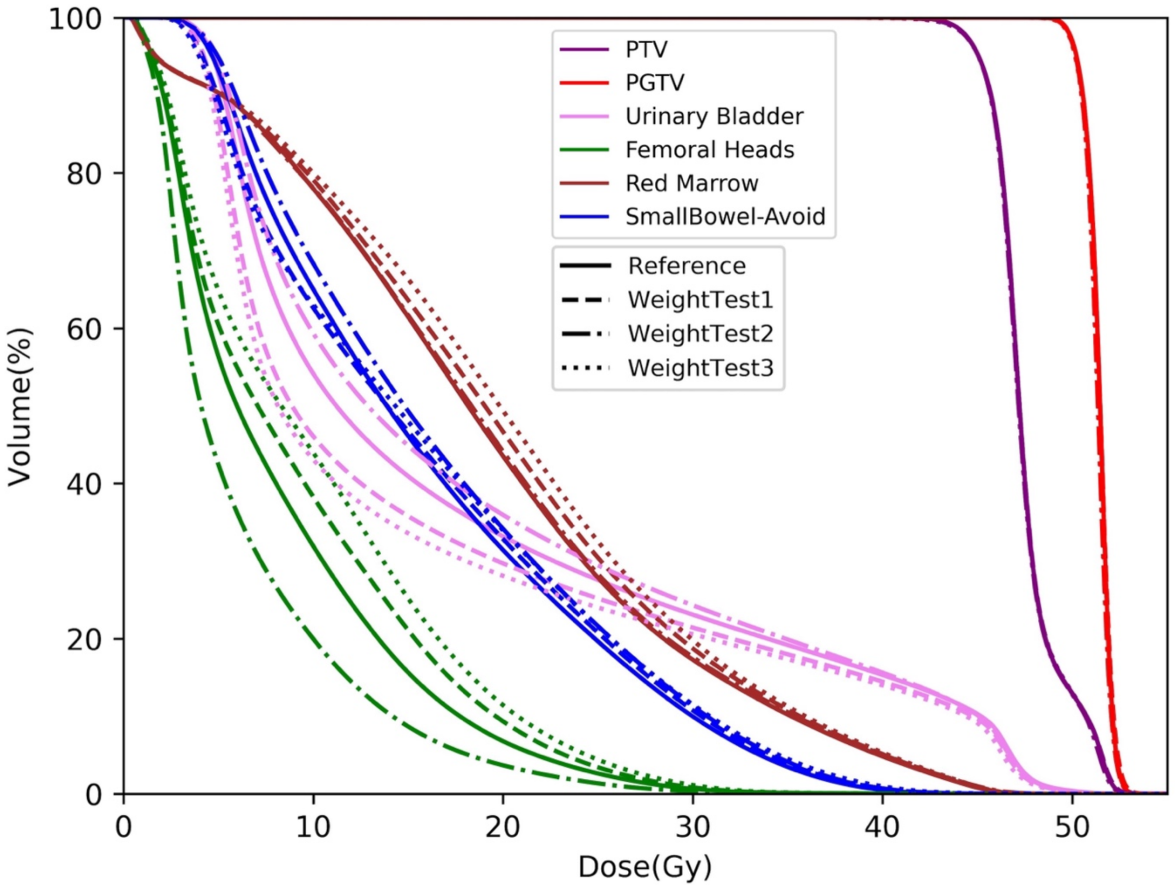

Figure 2 Average DVH comparisons between the Reference plans and WeightTest plans with different initial weight parameters

Using the proposed AIP system, automatic plans were successfully generated for different test groups, namely WeightTest1, WeightTest2, and WeightTest3. The average planning times for these groups were 36.8 ± 8.5 minutes, 38.6 ± 6.4 minutes, and 36.6 ± 7.7 minutes, respectively. The changes in the initial weight parameters did not have a significant impact on the planning time. In terms of plan quality, comparisons of the average DVH between the Reference plans and the WeightTest plans revealed that increasing the weight parameter can lead to better achievement of the corresponding objective. However, this improvement may come at the expense of other conflicting objectives. As the weight parameter continues to increase, the effect of improvement becomes weaker due to the constraints imposed by the target volume.

Based on these findings, it can be concluded that the initial weight parameters can be utilized to adjust the trade-offs between OARs in automatic plans to align with the preferences of different planners. This flexibility allows for customization and optimization based on individual requirements and constraints.

2. Plan quality changes during the optimization iteration

During the optimization process, the parameters highlighted with asterisks in Table 1 are automatically adjusted using an iterative optimization algorithm (IOA). To offer a detailed insight into the optimization process, Table S3

presents a numerical example of the iterative process, demonstrating the adjustments of parameters using the IOA method and the improvement of dose metrics. In the last iteration, the PlanNormalizationValue was applied to the plan when it passed. In addition, Figure S3 illustrates the DVHs of the plan in the initial three iterations and the final iteration, demonstrating a continuous improvement in plan quality as the iterative optimization progresses. This improvement is evident in the reduction of dose to the OAR, driving the optimization process towards meeting the specified dose requirements for the target volume. Initially, the automatic planning primarily emphasizes reducing the dose to the OAR, with corresponding adjustments decreasing as the impact on the OAR diminishes. In the final iteration, the automatic planning focuses on refining the dose distribution within the target volume.

Table S3 A numerical example of iterative optimization algorithm process

Iterations	Structures	Plan Objectives		Optimization Results			
		Dose (Gy)	Weight	Achieved Dose (Gy)	Diff (Gy)	pseudo-gradient (g)	ΔDose(Gy) / ΔWeight
1	PTV	46.00	200	44.67	-0.33	-	33
	PGTV	51.00	200	50.04	0.04	-	-4
	CTV	46.00	200	43.47	-1.53	-	100
	GTV	51.00	200	49.71	-0.29	-	29
	body	51.00	500	53.33	-0.33	-	33
	IrradVolume	47.00	200	49.26	-0.76	-	76
	urinary bladder	18.00	200	19.16	19.16	1	-2
	femoral heads	9.00	200	9.51	9.51	1	-2
	bone marrow	18.00	200	19.96	19.96	1	-2
	SmallBowel-Avoid	19.00	200	19.76	19.76	1	-2
2	PTV	46.00	232	44.22	-0.78	-	78
	PGTV	51.00	196	49.7	-0.3	-	30
	CTV	46.00	300	43.54	-1.46	-	100
	GTV	51.00	228	49.65	-0.35	-	35
	body	51.00	532	53.41	-0.41	-	41
	IrradVolume	47.00	276	48.39	0.11	-	-11
	urinary bladder	16.00	200	18.51	18.51	0.33	-1.15
	femoral heads	7.00	200	8.31	8.31	0.6	-1.67
	bone marrow	16.00	200	19.42	19.42	0.27	-0.99
	SmallBowel-Avoid	17.00	200	18.6	18.6	0.58	-1.64
3	PTV	46.00	309	44.36	-0.64	-	64
	PGTV	51.00	225	49.9	-0.1	-	10
	CTV	46.00	400	44.47	-0.53	-	53
	GTV	51.00	262	49.9	-0.1	-	10
	body	51.00	573	52.96	0.04	-	-4
	IrradVolume	47.00	265	48.76	-0.26	-	26
	urinary bladder	14.86	200	18.72	18.72	-0.18	0
	femoral heads	5.34	200	8.01	8.01	0.18	-0.58
	bone marrow	15.02	200	19.78	19.78	-0.37	0
	SmallBowel-Avoid	15.36	200	18.3	18.3	0.18	-0.57
4	PTV	46.00	372	44.45	-0.55	-	55
	PGTV	51.00	234	49.7	-0.3	-	30
	CTV	46.00	453	44.6	-0.4	-	40
	GTV	51.00	271	49.65	-0.35	-	35
	body	51.00	569	53.3	-0.3	-	30
	IrradVolume	47.00	290	48.75	-0.25	-	25
	urinary bladder	14.86	200	18.37	18.37	0	0
	femoral heads	4.77	200	7.94	7.94	0.12	-0.14
	bone marrow	15.02	200	19.49	19.49	0	0

Iterations	Structures	Plan Objectives		Optimization Results			
		Dose (Gy)	Weight	Achieved Dose (Gy)	Diff (Gy)	pseudo-gradient (g)	ΔDose(Gy) / ΔWeight
	SmallBowel-Avoid	14.80	200	17.82	17.82	0.85	-0.53
5 (PlanEvaluation = Pass, PlanNormalizationValue = 101%)	PTV	46.00	427	45.00	-	-	-
	PGTV	51.00	264	50.40	-	-	-
	CTV	46.00	493	45.10	-	-	-
	GTV	51.00	306	50.35	-	-	-
	body	51.00	598	53.71	-	-	-
	IrradVolume	47.00	314	49.11	-	-	-
	urinary bladder	14.86	200	18.88	-	-	-
	femoral heads	4.64	200	8.05	-	-	-
	bone marrow	15.02	200	19.82	-	-	-
	SmallBowel-Avoid	14.27	200	18.07	-	-	-

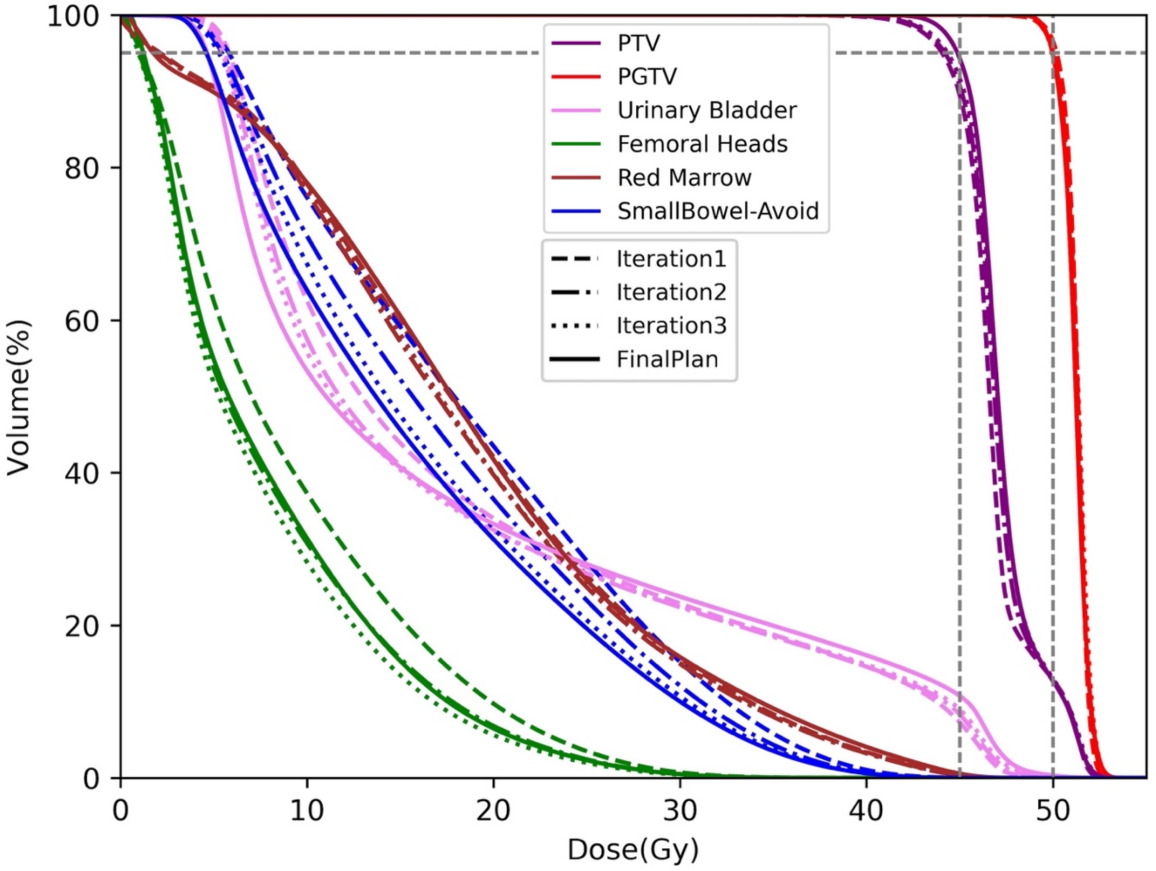

Figure 3 The change of automatic plan DVH during plan optimization, where the intersection of the gray dotted lines represents the prescription requirements.

3. Application of automatic iterative planning in nasopharyngeal carcinoma

The applicability of automatic planning in rectal cancer has been previously validated. To further evaluate the applicability of automated planning in complicated situations, we conducted an additional experimental test for

nasopharyngeal carcinoma (NPC). Specifically, the planning process in NPC involves considering 14 OARs and 35 objectives.

3.1 Patient cohort

A cohort of 16 NPC patients previously treated with two-arc coplanar VMAT in 2021 was retrospectively selected from the clinical database. The Computed Tomography scanning range was defined from the superior border of the second lumbar vertebra to the perineum, with a 3-mm slice thickness. The target structures included the gross tumor volume (GTV), the clinical target volume (CTV), and the lymph node volume (GTVnd). Subsequently, a uniform 5-mm margin was added to create the planning gross tumor volume (PGTV), the planning target volume (PTV), and the planning lymph node volume (PGTVnd) respectively. The considered OARs included the brainstem, the lens, the optic chiasma, the optic nerve, the oral cavity, the parotid gland, the middle ear, the mandible, the spinal cord, the thyroid gland, the larynx, and the temporal lobe. The clinical protocol defined a 33-fraction regimen with the SIB-VMAT technique, where 60 Gy, 70 Gy, and 70 Gy were prescribed to the 95% volume of the PTV, PGTV, and the PGTVnd, respectively.

3.2 Planning Template

Table S4 shows the initial parameter template for the automatic VMAT planning for NPC. two fixed objectives were also included in the template: the normal tissue objective with a fixed weight of 300 to control the dose spillage, and the monitor unit (MU) objective where the minimum MU, the maximum MU, and the penalizing weight were set at 600, 800 and 100, respectively.

Table S4 Plan template of NPC SIB-VMAT automatic iterative planning

number	Structure	Type	Plan Objectives			Ideal Objectives		Amplitude Conversion Factor	Initial Adjustment Amplitude (Gy)
			Volume (%)	Dose (Gy)	Weight	Volume (%)	Dose (Gy)		
1	PTV	Upper	0	74	400*	0	76	60	-
2	Body	Upper	0	74	400*	0	76	60	-
3	PTV	Lower	100	62	400*	95	60	100	-
4	CTV	Lower	100	62	400*	100	60	100	-
5	PGTV	Lower	100	72	400*	95	70	100	-
6	GTV	Lower	100	72	400*	100	70	100	-
7	PGTVnd	Lower	100	72	400*	95	70	100	-
8	GTVnd	Lower	100	72	400*	100	70	100	-
9	IrradVolume	Upper	0	64	300*	1	66	30	-
10	IrradVolume	Lower	100	63	300*	95	60	30	-
11	AvoidGapM	Upper	0	32*	300	0	0	-	1
12	AvoidGapA	Upper	0	45*	250	0	0	-	1
13	AvoidGapA	Mean	-	30*	250	-	0	-	2
14	AvoidGapP	Upper	0	30*	300	0	0	-	1.5
15	Brainstem	Upper	0	35*	300	0	0	-	1
16	Brainstem	Mean	-	34*	200	-	0	-	4
17	Brainstem+3mm	Upper	0	35*	300	0	0	-	1
18	Larynx	Mean	-	30*	150	-	0	-	2
19	Lens_L	Upper	0	2*	350	0	0	-	1
20	Lens_R	Upper	0	2*	350	0	0	-	1

number	Structure	Type	Plan Objectives			Ideal Objectives		Amplitude Conversion Factor	Initial Adjustment Amplitude (Gy)
			Volume (%)	Dose (Gy)	Weight	Volume (%)	Dose (Gy)		
21	Lens+3mm	Upper	0	3*	300	0	0	-	1
22	Mandible	Mean	-	36*	100	-	0	-	2
23	MidEar_L	Mean	-	35*	100	-	0	-	1.5
24	MidEar_R	Mean	-	35*	100	-	0	-	1.5
25	OpticChiasma	Upper	0	40*	300	0	0	-	2
26	OpticNerve_L	Upper	0	40*	280	0	0	-	2
27	OpticNerve_R	Upper	0	40*	280	0	0	-	2
28	OralCavity	Mean	0	33*	250	-	0	-	1.5
29	Parotid_L	Mean	-	20*	375	-	0	-	1
30	Parotid_R	Mean	-	20*	375	-	0	-	1
31	SpinalCord	Upper	-	25*	350	0	0	-	2
32	SpinalCord+5mm	Upper	-	30*	400	0	0	-	2
33	TemporalLobe_L	Mean	-	15*	150	-	0	-	1
34	TemporalLobe_R	Mean	-	15*	150	-	0	-	1
35	ThyroidGland	Mean	-	45*	150	-	0	-	1.5

3.3 Results

Using the proposed AIP system, automatic plans were generated for 16 cases of NPC patients, with an average time of 35.8 ± 10.6 minutes. Figure S4 presents a comparison of the DVH between the manual plans and automatic plans. Notably, there was no significant difference in DVH between automatic and manual planning in terms of dose control within the target volume. For OAR protection, the automatic planning approach successfully reduced the dose in regions such as the optic chiasm, brainstem, and spinal cord, without compromising other OARs. The statistical results in Table S4 further revealed that most OARs benefitted better sparing in automatic plans, except for a single OAR structure (thyroid gland). The automatic plans significantly reduced the max dose of optic nerve, optic chiasma, and larynx. These results collectively demonstrate that automatic planning can achieve a level of quality comparable to manual plans for NPC cases. More importantly, the automatic plans exhibited more consistent DVH metrics compared to the MP, due to identical trade-off preferences for all the patients. Automatic planning reduces the variance for most dosimetric metrics with significant differences in Table S5, such as the optic nerve D_{max}, the right parotid

D_{mean}, and the Larynx D_{max}. Consequently, our AIP system proves to be suitable for complex treatment sites.

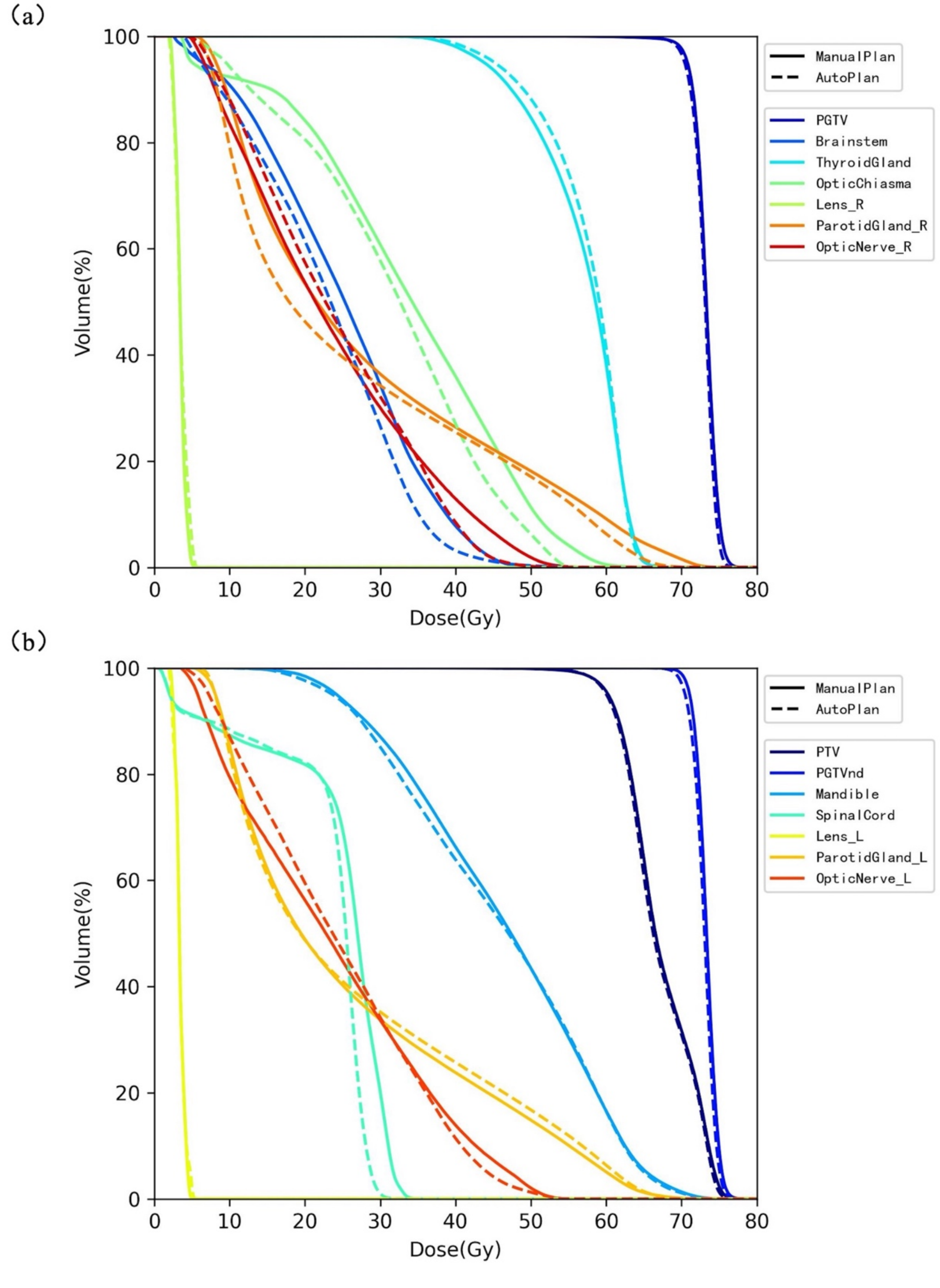

Figure 4 Average DVH comparisons between manual plans and automatic plans of nasopharyngeal carcinoma

Table S5 Dosimetric metrics statistics of manual plans and automatic plans for NPC SIB-VMAT

ROI	Metrics	Manual plans	Automatic plans	p-value
PTV	Dmax(Gy)	78.07 (±0.85)	78.11 (±0.28)	0.86

ROI	Metrics	Manual plans	Automatic plans	p-value
	D98%(Gy)	58.31 (±0.78)	58.19 (±0.21)	1
	D95%(Gy)	60.34 (±0.59)	60.03 (±0.02)	0.05
	CI	0.81 (±0.02)	0.80 (±0.02)	**0.03**
	HI	0.19 (±0.02)	0.18 (±0.01)	0.12
PGTV	$D_{95\%}$(Gy)	70.84 (±0.38)	70.43 (±0.21)	**0.004**
	CI	0.46 (±0.11)	0.47 (±0.13)	0.31
	HI	0.08 (±0.02)	0.08 (±0.01)	0.66
PGTVnd	$D_{95\%}$(Gy)	71.13 (±0.41)	70.50 (±0.23)	**<0.001**
	CI	0.22 (±0.05)	0.22 (±0.05)	0.70
	HI	0.07 (±0.01)	0.08 (±0.00)	**0.006**
Brainstem	D_{max} (Gy)	44.93 (±5.32)	45.72 (±6.58)	0.86
OpticNerve_L	D_{max} (Gy)	49.86 (±9.59)	46.15 (±8.09)	**0.008**
OpticNerve_R	D_{max} (Gy)	49.30 (±9.41)	45.09 (±8.17)	**0.002**
OpticChiasma	D_{max} (Gy)	56.68 (±8.34)	50.09 (±9.37)	**0.001**
Lens_L	D_{max} (Gy)	4.15 (±0.71)	4.12 (±0.74)	1
Lens_R	D_{max} (Gy)	4.21 (±0.73)	4.26 (±0.91)	0.66
Mandible	D_{mean} (Gy)	46.39 (±3.93)	45.78 (±3.24)	0.34
SpinalCord	D_{max} (Gy)	32.24 (±1.56)	31.00 (±1.80)	0.10
Parotid_L	D_{mean} (Gy)	26.32 (±3.55)	26.71 (±3.71)	0.19
Parotid_R	D_{mean} (Gy)	28.55 (±10.01)	26.01 (±7.90)	**0.04**
Larynx	D_{mean} (Gy)	46.48 (±2.00)	46.17 (±1.53)	0.33
	D_{max} (Gy)	73.20 (±2.30)	72.70 (±1.72)	**0.05**
ThyroidGland	D_{mean} (Gy)	56.73(±2.50)	57.36 (±1.92)	0.34
MU	-	763.5 (±51.8)	795.3 (±26.2)	**0.02**
